# Host gene expression analysis in Sri Lankan melioidosis patients

**DOI:** 10.1371/journal.pntd.0005643

**Published:** 2017-06-19

**Authors:** Shivankari Krishnananthasivam, Nimanthi Jayathilaka, Harindra Darshana Sathkumara, Enoka Corea, Mohan Natesan, Aruna Dharshan De Silva

**Affiliations:** 1 Genetech Research Institute, Colombo, Sri Lanka; 2 Department of Chemistry, Faculty of Science, University of Kelaniya, Kelaniya, Sri Lanka; 3 Department of Microbiology, Faculty of Medicine, University of Colombo, Colombo, Sri Lanka; 4 Molecular and Translational Sciences, United States Army Medical Research Institute of Infectious Diseases, Frederick, MD, United States of America; 5 Division of Vaccine Discovery, La Jolla Institute of Allergy and Immunology, La Jolla, CA, United States of America; University of California San Diego School of Medicine, UNITED STATES

## Abstract

**Background:**

Melioidosis is a life threatening infectious disease caused by the gram-negative bacillus *Burkholderia pseudomallei* predominantly found in southeast Asia and northern Australia. Studying the host transcription profiles in response to infection is crucial for understanding disease pathogenesis and correlates of disease severity, which may help improve therapeutic intervention and survival. The aim of this study was to analyze gene expression levels of human host factors in melioidosis patients and establish useful correlation with disease biomarkers, compared to healthy individuals and patients with sepsis caused by other pathogens.

**Methods:**

The study population consisted of 30 melioidosis cases, 10 healthy controls and 10 sepsis cases caused by other pathogens. Total RNA was extracted from peripheral blood mononuclear cells (PBMC’s) of study subjects. Gene expression profiles of 25 gene targets including 19 immune response genes and 6 epigenetic factors were analyzed by real time quantitative polymerase chain reaction (RT-qPCR).

**Principal findings:**

Inflammatory response genes; TLR4, late onset inflammatory mediator HMGB1, genes associated with antigen presentation; MICB, PSMB2, PSMB8, PSME2, epigenetic regulators; DNMT3B, HDAC1, HDAC2 were significantly down regulated, whereas the anti-inflammatory gene; IL4 was up regulated in melioidosis patients compared to sepsis cases caused by other pathogens. Septicaemic melioidosis cases showed significant down regulation of IL8 compared to sepsis cases caused by other pathogens. HMGB1, MICB, PSMB8, PSMB2, PSME2, HDAC1, HDAC2 and DNMT3B showed consistent down regulation of gene expression in melioidosis patients compared to other sepsis infection, irrespective of comorbidities such as diabetes, duration of clinical symptoms and antibiotic treatment.

**Significance:**

Specific immune response genes and epigenetic regulators are differentially expressed among melioidosis patients and patients with sepsis caused by other pathogens. Therefore, these genes may serve as biomarkers for disease diagnosis to distinguish melioidosis from cases of sepsis due to other infections and therapeutic intervention for melioidosis.

## Author summary

Melioidosis is a life threatening infectious disease caused by a soil-associated gram-negative bacterium, *B*. *pseudomallei*. Melioidosis is endemic in southeast Asia and northern Australia; however, the global distribution of *B*. *pseudomallei* and the disease burden of melioidosis is still poorly understood. Melioidosis is severely under-reported in several tropical countries in which it is probably endemic and warrants a public health response. A recent research article predicts the global melioidosis burden to be 165 million cases with a predicted 73 million cases from the high risk zone of south Asia. Melioidosis is difficult to treat as *B*. *pseudomallei* is resistant to many antibiotics and requires a long course of treatment. Mortality rate remains high in endemic areas with reoccurrence being common. Therefore, it is imperative to diagnose the disease at an early stage and provide vital clinical care to reduce the mortality rate. With limitations in treatment and lack of a vaccine, it is crucial to study the immune response mechanisms to this infection to get a better understanding of disease pathogenesis and susceptibility. Therefore, this study aimed to analyze the gene expression levels of important immune response genes and epigenetic modifiers to establish useful correlation for diagnostic and therapeutic purposes.

## Introduction

Melioidosis, an emerging infectious disease of public health importance in many tropical countries, is caused by the gram negative bacterium *Burkholderia pseudomallei* and is commonly reported in southeast Asia and northern Australia [1]. *B*. *pseudomallei* is an intracellular facultative pathogen, which is widely distributed in muddy soils such as rice paddy fields and pooled surface water in endemic regions [2]. Skin inoculation is considered as the main route of infection. However, evidence also suggests that inhalation of aerosolized bacteria during extreme weather events such as cyclones, heavy rainfall, storms and ingestion of bacteria via contaminated water are important routes of infection [2, 3]. The disease is strongly associated with comorbidities such diabetes mellitus, chronic kidney disease, thalassemia, immunosuppression and excessive alcohol intake [1, 4, 5]. Melioidosis has a wide spectrum of severity ranging from acute to chronic forms of illness, with common clinical presentations being pneumonia, septicaemia, abscesses and skin lesions [1, 5]. There are no available vaccines for disease prevention. Melioidosis is challenging to treat as *B*. *pseudomallei* is intrinsically resistant to many antibiotics and mortality in endemic regions is high (40–50%)[2]. Recurrence due to reactivation of latent infection is also common [2]. However, standard guidelines for therapy have proven effective in reducing mortality and preventing recurrences [6, 7], with early diagnosis playing a crucial role in successful treatment. Therefore, development of new early diagnostic tools and therapeutic strategies is imperative [2].

Studying the host immune responses to infection is crucial for understanding disease pathogenesis, susceptibility to severe disease and immune correlates of protection. Gene expression profiling of important host factors from peripheral blood, which constitutes an accessible source of circulating immune cells, provides key insights into host responses and defense mechanisms against infection[8]. Several studies have shown an association between expression levels of cytokines and disease progression and severity [9–13]. Further evaluation of gene expression at the mRNA level can help us to establish useful disease biomarkers and immune correlates of *B*. *pseudomallei* infection.

Toll-like receptors (TLR) play a major role in host defense, as they detect host invasion by pathogens and initiate immune responses that form the crucial link between innate and adaptive immune responses, thus influencing disease progression[14, 15]. Several studies have suggested that TLR’s play a significant role in host susceptibility to melioidosis [13, 16–18]. A broader study to determine TLR receptor expression and signaling pathways could provide insights for understanding disease susceptibility and progression following *B*. *pseudomallei* infection.

Major histocompatibility complexes (MHC) and genes associated with antigen presentation pathways play an important role in host defense mechanisms against intracellular pathogens. Gene expression profiling studies on melioidosis also suggest that proteasomes associated with antigen presentation pathways have an important role in disease progression and severity[8].

Epigenetic modifications, such as DNA methylation and histone modifications are regulatory mechanisms which have a considerable impact on gene transcription and, thereby, pathophysiological processes leading to altered risk for disease [19]. Several studies have shown that the enzymes responsible for these epigenetic modifications are dysregulated in several common diseases, playing an influential role in initiation and progression of the disease by modulating host immune responses, inflammation and intracellular host defenses [19–25]. Studying these epigenetic regulators which have already been implicated in host response to infection by pathogens like bacteria and viruses, can facilitate better understanding of the molecular basis of disease pathogenesis and susceptibility to severe melioidosis[24].

Diagnosing melioidosis based on clinical symptoms from a broad range of infection and septicaemic conditions can be challenging, thus requires tedious, time consuming laboratory diagnostic tests for disease confirmation. Such laboratory facilities may not be available in rural hospital settings and rapid diagnostic point of care tests would be very useful in early diagnosis of infection, provided diagnostic biomarkers for melioidosis are identified. Therefore, studies comparing host immune responses during melioidosis and septicaemic conditions caused by other pathogens, could potentially identify differentially expressed immune markers to serve as diagnostic markers and in monitoring antibiotic treatment regime during therapeutic intervention.

In this study we aimed to analyze the gene expression profiles of important cytokines, TLR’s, genes associated with antigen presentation pathways and cell mediated immunity and epigenetic factors in melioidosis patients, patients with sepsis infection caused by other pathogens and healthy individuals in order to establish correlations with disease biomarkers. Our study on human host mRNA expression profile is the first reported study in a Sri Lankan melioidosis patient cohort and has identified significant differential expression of key immune response genes and epigenetic regulators during melioidosis infection.

## Methods

### Patient enrollment

Nationwide active surveillance for melioidosis was established in multiple state and private hospitals throughout Sri Lanka, with ethics approval from the Ethics Review Committee, Faculty of Medicine, University of Colombo, Sri Lanka and Office of Human Use and Ethics (OHU&E) of U.S. Army Medical Research Institute of Infectious Diseases (USAMRIID); and U.S. Army Medical Research and Material Command- Office of Research Protection- Human Research Protection Office (USAMRMC-ORP-HRPO). Patients fitting the clinical case definition of melioidosis i.e. febrile illness for more than 5 days, pneumonia, septic arthritis, skin lesions, septicemia, lung, soft tissue or deep abscess were recruited for initial screening for melioidosis with informed consent. Blood, pus and other patient specimens were collected for bacterial cultures and serum samples were collected for the indirect haemagglutination (IHA) antibody test. Any positive bacterial cultures were further screened and confirmed as *B*. *pseudomallei* by PCR. All samples for the study were collected between September 2014 and December 2015.

Patients who were culture positive for *B*. *pseudomallei* and / or had high antibody (>640) titers by the IHA test were recruited for our study with written informed consent and classified as cases of melioidosis. Culture positive samples were considered as confirmed cases of melioidosis. Samples with an antibody titre of >640 by IHA testing were considered as probable cases of melioidosis.

We also recruited healthy individuals and patients fitting the clinical definition of severe sepsis/septic shock (as per the 2012 International Guidelines for Management of Severe Sepsis and Septic shock) who were negative for *B*. *pseudomallei*, as controls for our gene expression profiling study[26].

### Bacterial culture and identification

Primary isolation was done at the admitting hospital and relied on conventional culture techniques for blood, sputum, pus and other specimens. Bacterial isolates that were oxidase positive, gentamicin-resistant and gram-negative bacilli were forwarded to the reference laboratory in Colombo where they were sub-cultured to establish pure growth and maintained at −70°C in 15% brain heart infusion (BHI) glycerol for subsequent definitive tests. Bacteria were resuscitated by subculture onto 5% blood agar and incubated for 24 h at 37°C to give single colony growth for all subsequent tests.

### Real time PCR assay for confirmation of *B*. *pseudomallei*

A single colony of *B*. *pseudomallei* grown on blood agar from patients sample was re-suspended in ultrapure water. The suspension was heated at 95°C for 10 min and centrifuged at 13500 x g to pellet the cell debris. The supernatant was used as the template for all subsequent PCR assays. Real time PCR assay was done for gene targets of the lpxO, YLF and BTFC gene clusters using the primers and methods described in Merritt et al, 2006 and Tuanyok et al, 2007 respectively [27, 28].

### IHA antibody testing

Antibody testing against *B*. *pseudomallei* antigen was performed using an in-house method adapted from Alexander et al.1970. Antigen was prepared from a heat killed culture supernatant of a Sri Lankan *B*. *pseudomallei* strain BPs7. A 1/80 diluted antigen prepared was used to sensitize sheep erythrocytes. Serum samples were heat inactivated at 56°C for 30 minutes and tested by serial dilution from 1/10 to 1/10,240 with sensitized sheep erythrocytes and the highest dilution at which hemagglutination occurred was recorded as end point titer [29].

### Sample collection and processing

10 ml of whole blood was collected from patients/volunteers after informed consent, of which 7ml were collected into B.D vacutainer mononuclear cell preparation tubes (catalog no:362761) for lymphocyte purification and 3ml were collected into BD vacutainer EDTA tube for plasma collection. The lymphocytes were purified using the Ficoll fractionation method as per manufacturer’s instructions and lysed with RLT buffer (Qiagen RNeasy mini kit-catalog no: 74104), homogenized and stored at -80°C for total RNA extraction.

### Total RNA extraction and cDNA synthesis

Total RNA was extracted from the stored cell lysate samples using the Qiagen RNeasy mini kit (catalog no:74104) as per manufacturer protocol. 30μl of eluted RNA was stored at -80°C until further use. RNA extracted from 2 million PBMC’s was used for cDNA synthesis as the standard for all samples analyzed by RT-qPCR. cDNA synthesis for a 25μl reaction was carried out with 2μl of Qiagen genomic DNA wipeout buffer (catalog no:205311), according to manufacturer’s recommendations and incubated for 10 minutes at 42°C. 0.5μl each of Promega random primers (catalog C1181) and Promega oligo dT 15 primers (catalog no: C1101) was added to the reaction and incubated at 70°C for 5 minutes. A separate 10 μl reaction mixture containing 5μl Promega M-MLV 5X Reverse transcriptase buffer-, 1.25μl 10mM Promega dNTP’s -, 0.5μl Promega RNasin Plus RNase inhibitor-, 2.25μl nuclease free water- and 1μl Promega M-MLV reverse transcriptase- was prepared and added to the earlier reaction mixture (DNA wipeout reaction) and incubated at 37°C for 1 hour, followed by 95°C for 3 minutes. The synthesized cDNA samples were stored at -20°C until further use.

### Real time qPCR and gene expression analysis

The primers for the gene expression analysis study (Table 1, S2 Table) were verified by real time PCR followed by agarose gel visualization for correct amplicon sizes. PCR efficiency calculation by standard curve efficiency calculations was performed in triplicates for each primer pair to ascertain PCR efficiency.

**Table 1:** Details of gene targets for gene expression analysis.

Abbreviated name of gene target	Full Name of gene target
^a^GAPDH	Glyceraldehyde 3-phosphate dehydrogenase
^a^18S rRNA	18S ribosomal RNA
^b^PLCE1	1-Phosphatidylinositol-4,5-bisphosphate phosphodiesterase epsilon-1
IL1β	Interleukin 1 beta
IL4	Interleukin 4
IL6	Interleukin 6
IL8	Interleukin 8
IL10	Interleukin 10
IL12	Interleukin 12
IL15	Interleukin 15
IL18	Interleukin 18
CCL5	Chemokine (C-C motif) ligand 5 /RANTES.
IFNγ	Interferon gamma
TNFα	Tumor necrosis factor alpha
HMGB1	High mobility group box 1 protein /high-mobility group protein 1 (HMG-1)
TLR2	Toll-like receptor 2
TLR4	Toll-like receptor 4
MICB	MHC class I polypeptide-related sequence B
PSMB8	Proteasome subunit beta type-8 /20S proteasome subunit beta-5i
PSMB2	Proteasome subunit beta type-2 / 20S proteasome subunit beta-4
PSME2	Proteasome activator complex subunit 2
PSMA5	Proteasome subunit alpha type-5 / 20S proteasome subunit alpha-5
HLADMB	HLA class II histocompatibility antigen, DM beta chain
DNMT1A	DNA methyltransferase 1A
DNMT3A	DNA (cytosine-5)-methyl transferase 3A
DNMT3B	DNA (cytosine-5-)-methyl transferase 3 beta
HDAC1	Histone deacetylase 1
HDAC2	Histone deacetylase 2
HDAC4	Histone deacetylase 4

^a^GAPDH and 18S rRNA primers are house keeping genes.

^b^PLCE1 primer pair used as genomic DNA control to amplify genomic region of PLCE1.

Real Time qPCR was performed in a 25μl reaction mixture containing 12.5μl of RT^2^ SYBR green ROX qPCR master mix (catalog no: 330520), 1μl each of 10uM forward and reverse primers, 9.5μl of nuclease free water and 1μl of cDNA template, on the Qiagen Q5 plex thermal cycler. The PCR conditions were 94°C-2 min of initial denaturation followed by 35 cycles of 94°C-30 sec, 63°C-1 min, 72°C-1 min.

30 melioidosis cases (identified as confirmed or probable cases), 10 healthy negative controls and 10 sepsis negative controls (negative for *B*. *pseudomallei*) were analyzed by RT-qPCR. Housekeeping genes GAPDH and 18S rRNA, negative controls with no template, genomic DNA control and 25 target genes were run in duplicates for all samples analyzed.

### Data analysis

Efficiency corrected relative expression was calculated according to Pfaffl, 2001 [30]. 18S rRNA housekeeping gene was used for normalizing the data for analysis, as it was stably expressed across experimental and control samples. Efficiency corrected delta delta Ct values / relative expression are presented as log values to the base 2. The statistical analysis was performed using SAS PROC MIXED, version 9.4 with statistical significance of relative expression ratios obtained by paired T tests. Values of P<0.05 were considered statistically significant.

## Results

A total of 30 patient samples classified as cases of melioidosis were analyzed of which 23 were confirmed cases (culture positive) and 7 were probable cases (high antibody titre positive). Among the 23 confirmed cases of melioidosis, 18 were classified as septicaemic or bacteriaemic. A total of 27 identified positive cases had comorbidities such as diabetes, alcoholism, kidney disease etc. Healthy volunteers and cases of sepsis (negative for *B*. *pseudomallei*) of 10 each, were also profiled as negative controls for the study.

We analyzed our data based on duration of clinical symptoms, duration of antibiotics and associated comorbidities as these listed factors play a confounding role in host gene expression during infection. Such an analysis was needed to understand host gene expression during early and late acute phases of infection and disease severity in susceptible groups.

Interestingly, we did not find significant differential expression of immune response genes and epigenetic modifiers in melioidosis patients compared to healthy controls. Cases of sepsis due to other infections, on the other hand, showed statistically significant differential gene expression from the melioidosis patients for most of the genes investigated.

### Gene expression profile of cytokines

**Fig 1:**
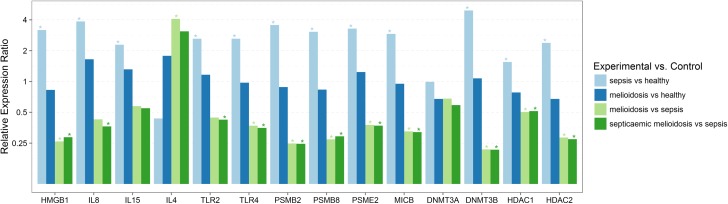
Relative expression of genes involved in immune response and epigenetic regulation in melioidosis patients compared to patients with sepsis infections caused by other pathogens and healthy controls. Statistically significant differential expression of genes in PBMC’s from melioidosis patients (n = 30), septicaemic melioidosis patients (n = 18) compared to sepsis controls (n = 10). Significant differential expression was not observed among melioidosis patients compared to healthy controls (n = 10) while significant differential expression was observed when compared to sepsis controls. Expression levels were normalized against 18SrRNA. Relative expression ratio based on efficiency corrected delta delta Ct values presented as log values to the base 2, >1.5 considered as up regulation and ≤0.5 considered as down regulation, with P<0.05 considered statistically significant.

mRNA expression levels of IL18, CCL5 and IL12 in PBMC’s were low in all the samples. IL8, IL15 and HMGB1 were significantly up regulated in other sepsis cases compared to healthy controls (Table 2, Fig 1).

**Table 2:** mRNA expression in PBMC’s of melioidosis patients (n = 30) compared to other sepsis cases (n = 10) and healthy negative controls (n = 10).

	Melioidosis vs Healthy controls		Melioidosis vs Sepsis controls		Sepsis cases vs Healthy controls	
Gene	Relative expression ratio [95% CI]	P value	Relative expression ratio [95% CI]	P value	Relative expression ratio [95% CI]	P value
HMGB1	0.83[0.444,1.539]	0.5324	0.26[0.132,0.510]*	0.0005	3.18[1.539,6.578]*	0.0036
IL6	1.56[0.670,3.623]	0.2912	0.89[0.360,2.189]	0.7867	1.76[0.710,4.344]	0.2079
IL8	1.65[0.727,3.733]	0.2187	0.43[0.169,1.080]	0.0699	3.86[1.411,10.540]*	0.0114
IL1β	1.33[0.554,3.205]	0.5005	0.77[0.258,2.320]	0.6254	1.72[0.516,5.738]	0.3545
IFNγ	1.34[0.826,2.189]	0.2174	0.88[0.496,1.572]	0.6522	1.52[0.800,2.899]	0.1864
TNFα	1.22[0.705,2.124]	0.4573	0.81[0.408,1.598]	0.5173	1.52[0.741,3.100]	0.2365
IL15	1.31[0.751,2.296]	0.3254	0.57[0.258,1.279]	0.1610	2.29[1.025,5.103]*	0.0443
IL4	1.78[0.115,27.531]	0.3533	4.09[1.178,14.173]*	0.0366	0.44[0.065,2.942]	0.2329
TLR2	1.16[0.666,2.034]	0.5844	0.44[0.196,1.009]	0.0522	2.62[1.182,5.803]*	0.0212
TLR4	0.97[0.532,1.777]	0.9247	0.37[0.141,0.974]*	0.0448	2.62[1.025,6.700]*	0.0450
MICB	0.95[0.533,1.699]	0.8607	0.33[0.183,0.582]*	0.0006	2.92[1.534,5.545]*	0.0026
HLADMB	0.84[0.502,1.396]	0.4814	0.69[0.396,1.204]	0.1809	1.21[0.682,2.156]	0.4897
PSMB2	0.88[0.513,1.519]	0.6403	0.25[0.121,0.508]*	0.0008	3.56[1.708,7.420]*	0.0021
PSME2	1.24[0.763,1.998]	0.3757	0.38[0.195,0.726]*	0.0061	3.28[1.684,6.389]*	0.0017
PSMB8	0.83[0.456,1.519]	0.5356	0.27[0.131,0.565]*	0.0014	3.06[1.443,6.480]*	0.0060
PSMA5	0.75[0.421,1.328]	0.3086	0.84[0.412,1.706]	0.6102	0.89[0.448,1.778]	0.7301
DNMT1A	0.66[0.376,1.171]	0.1494	0.51[0.211,1.213]	0.1171	1.31[0.542,3.169]	0.5224
DNMT3A	0.68[0.416,1.097]	0.1087	0.68[0.356,1.300]	0.2284	0.99[0.533,1.849]	0.9793
DNMT3B	1.07[0.636,1.814]	0.7770	0.22[0.088,0.539]*	0.0040	4.94[1.948,12.503]*	0.0031
HDAC1	0.78[0.556,1.095]	0.1441	0.50[0.353,0.719]*	0.0006	1.55[1.059,2.267]*	0.0266
HDAC2	0.68[0.377,1.212]	0.1799	0.28[0.126,0.642]*	0.0048	2.38[1.047,5.403]*	0.0398
HDAC4	0.99[0.588,1.652]	0.9549	0.64[0.322,1.270]	0.1880	1.54[0.786,3.025]	0.1911

PBMC, peripheral blood mononuclear cells; CI, confidence interval; Relative expression ratio based on efficiency corrected delta delta Ct values presented as log values to the base 2, >1.5 indicates up regulation and ≤0.5 indicates down regulation in the experimental group compared to control group

* indicates statistically significant differential expression where P-value <0.05, P values calculated by paired t-tests

**Fig 2:**
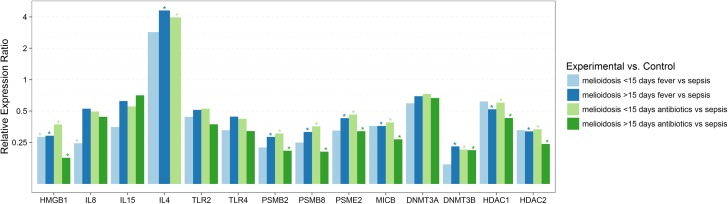
Relative expression of genes involved in immune responses and epigenetic regulation in melioidosis patients compared to patients with sepsis infections caused by other pathogens, in relation to duration of fever/clinical symptoms and antibiotics treatment. Differential gene expression in PBMC’s from melioidosis patients with ≤15 days of fever (n = 4), melioidosis patients with >15 days of fever (n = 25), melioidosis patients with ≤15 days of treatment with antibiotics (n = 15), melioidosis patients with >15 days of treatment with antibiotics (n = 12) compared to sepsis controls (n = 10), did not change significantly due to the duration of fever or duration of treatment with antibiotics. Expression levels were normalized against 18S rRNA. Relative expression ratio based on efficiency corrected delta delta Ct values presented as log values to the base 2, >1.5 considered up regulated and ≤0.5 considered as down regulation, with P<0.05 considered statistically significant.

**Fig 3:**
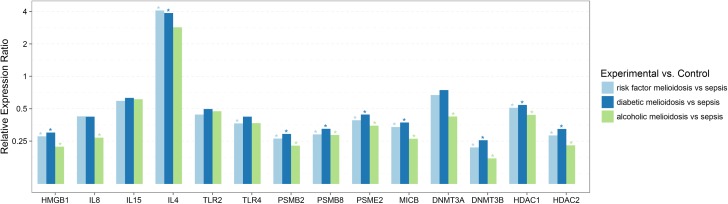
Relative expression of genes involved in immune responses and epigenetic regulation in melioidosis patients compared to patients with sepsis infections caused by other pathogens, in relation to associated comorbidities. Differential gene expression in PBMC’s from melioidosis patients with underlying commorbidities/risk factors (n = 27), alcoholic melioidosis patients (n = 8), diabetic melioidosis patients (n = 20), compared to sepsis controls (n = 10), did not change significantly due to associated commorbidities/risk factors for melioidosis. Expression levels were normalized against 18S rRNA. Relative expression ratio based on efficiency corrected delta delta Ct values presented as log values to the base 2, >1.5 was considered as up regulated and ≤0.5 was considered as down regulated, with P<0.05 considered statistically significant.

IL6, IL8, IFNγ, TNFα, IL1β and IL15 did not show any statistically significant differential gene expression in melioidosis samples compared to sepsis cases. IL4 showed significant up regulation in melioidosis cases compared to other sepsis cases (Table 2, Fig 1) while HMGB1, an inflammatory mediator was consistently down regulated in melioidosis cases compared to other sepsis cases, irrespective of all other factors like comorbidities, duration of fever/clinical symptoms and antibiotic treatment (Tables 2–5, Figs 1–3).

**Table 3:** mRNA expression in PBMC’s of septicaemic melioidosis patients (n = 18), melioidosis patients with underlying comorbidities/risk factors (n = 27), melioidosis with ≤15 days of fever (n = 4) compared to sepsis negative controls (n = 10).

	Septicaemic Melioidosis vs Sepsis controls		Melioidosis with risk factors vs Sepsis controls		Melioidosis (≤15 days of fever) vs Sepsis controls	
Gene	Relative expression ratio [95% CI]	P value	Relative expression ratio [95% CI]	P value	Relative expression ratio [95% CI]	P value
HMGB1	0.29[0.144,0.567]*	0.0011	0.28[0.140,0.548]*	0.0008	0.28[0.086,0.933]*	0.0414
IL6	0.84[0.350,2.030]	0.6902	0.84[0.331,2.133]	0.7042	0.20[0.000,2506.893]	0.3490
IL8	0.36[0.144,0.922] *	0.0348	0.42[0.164,1.097]	0.0743	0.25[0.068,0.885]*	0.0355
IL1β	0.58[0.186,1.785]	0.3167	0.73[0.243,2.221]	0.5611	0.35[0.060,1.989]	0.1942
IFNγ	0.82[0.457,1.472]	0.4816	0.89[0.496,1.588]	0.6681	1.01[0.363,2.789]	0.9885
TNFα	0.69[0.350,1.363]	0.2659	0.83[0.413,1.665]	0.5798	0.53[0.154,1.832]	0.2546
IL15	0.55[0.234,1.288]	0.1568	0.59[0.263,1.325]	0.1864	0.35[0.018,6.719]	0.3228
IL4	3.09[0.740,12.862]	0.0790	4.09[1.178,14.173]*	0.0366	2.86[0.833,9.802]	0.0893
TLR2	0.42[0.183,0.980]*	0.0452	0.44[0.191,1.017]	0.0543	0.44[0.075,2.579]	0.2843
TLR4	0.35[0.133,0.936]*	0.0378	0.37[0.137,0.974]*	0.0447	0.33[0.053,2.045]	0.1832
MICB	0.32[0.181,0.566]*	0.0005	0.34[0.187,0.612]*	0.0010	0.36[0.096,1.357]	0.1020
HLADMB	0.71[0.402,1.266]	0.2348	0.74[0.422,1.300]	0.2809	0.60[0.108,3.358]	0.4523
PSMB2	0.25[0.117,0.516]*	0.0009	0.26[0.128,0.543]*	0.0012	0.22[0.049,1.014]	0.0514
PSME2	0.37[0.186,0.739]*	0.0072	0.39[0.201,0.760]*	0.0084	0.32[0.058,1.812]	0.1468
PSMB8	0.29[0.138,0.615]*	0.0027	0.29[0.138,0.604]*	0.0022	0.25[0.058,1.072]	0.0582
PSMA5	0.72[0.349,1.465]	0.3400	0.83[0.400,1.715]	0.5958	0.80[0.241,2.647]	0.6419
DNMT1A	0.44[0.178,1.080]	0.0705	0.50[0.207,1.205]	0.1135	0.48[0.064,3.638]	0.3620
DNMT3A	0.59[0.301,1.151]	0.1149	0.67[0.346,1.300]	0.2228	0.59[0.156,2.245]	0.3298
DNMT3B	0.22[0.086,0.545]*	0.0040	0.22[0.088,0.543]*	0.0040	0.15[0.005,4.548]	0.1327
HDAC1	0.51[0.359,0.736]*	0.0009	0.51[0.355,0.733]*	0.0008	0.62[0.308,1.237]	0.1346
HDAC2	0.27[0.121,0.618]*	0.0039	0.28[0.123,0.646]*	0.0049	0.33[0.091,1.180]	0.0783
HDAC4	0.55[0.272,1.124]	0.0966	0.65[0.324,1.306]	0.2124	0.52[0.034,7.957]	0.4746

PBMC, peripheral blood mononuclear cells; CI, confidence interval; Relative expression ratio based on efficiency corrected delta delta Ct values presented as log values to the base 2, >1.5 indicates up regulation and ≤0.5 indicates down regulation in the experimental group compared to control group

* indicates statistically significant differential expression where P-value <0.05, P values calculated by paired t-tests

**Table 4:** mRNA expression in PBMC’s of melioidosis patients with >15 days of fever (n = 25), melioidosis patients with ≤15 days of antibiotics (n = 15), melioidosis patients with >15 days of antibiotics (n = 12) compared to other sepsis cases (n = 10).

	Melioidosis (>15 days fever) vs Sepsis controls		Melioidosis (≤15 days of antibiotics) vs Sepsis controls		Melioidosis (>15 days of antibiotics) vs Sepsis controls	
Gene	Relative expression ratio [95% CI]	P value	Relative expression ratio [95% CI]	P value	Relative expression ratio [95% CI]	P value
HMGB1	0.29[0.149,0.566] *	0.001	0.37[0.193,0.712]*	0.0053	0.18[0.065,0.493]*	0.0023
IL6	1.10[0.444,2.732]	0.8281	1.08[0.345,3.379]	0.8895	0.88[0.244,3.140]	0.8283
IL8	0.53[0.206,1.345]	0.1682	0.49[0.169,1.446]	0.1869	0.44[0.142,1.366]	0.1463
IL1β	0.94[0.310,2.851]	0.9071	0.98[0.299,3.221]	0.9744	0.71[0.202,2.467]	0.5660
IFNγ	0.91[0.507,1.630]	0.7333	0.97[0.518,1.805]	0.9116	0.72[0.375,1.396]	0.3133
TNFα	0.94[0.475,1.871]	0.8585	0.90[0.411,1.987]	0.7914	0.75[0.342,1.643]	0.4508
IL15	0.62[0.281,1.385]	0.2263	0.55[0.234,1.311]	0.1663	0.71[0.226,2.218]	0.5196
IL4	4.60[1.317,16.085]*	0.0299	3.95[1.132,13.800]*	0.0384	-	
TLR2	0.51[0.229,1.144]	0.0966	0.53[0.232,1.195]	0.1167	0.37[0.105,1.317]	0.1165
TLR4	0.44[0.171,1.144]	0.087	0.42[0.160,1.114]	0.0776	0.32[0.078,1.328]	0.1095
MICB	0.36[0.204,0.634]*	0.0012	0.39[0.221,0.687]*	0.0026	0.27[0.102,0.704]*	0.0108
HLADMB	0.79[0.464,1.347]	0.3671	0.84[0.483,1.444]	0.4997	0.58[0.234,1.446]	0.2246
PSMB2	0.28[0.139,0.571]*	0.0016	0.30[0.148,0.626]*	0.003	0.21[0.078,0.552]*	0.0032
PSME2	0.43[0.225,0.811]*	0.0128	0.46[0.238,0.899]*	0.0256	0.32[0.133,0.773]*	0.0140
PSMB8	0.31[0.153,0.646]*	0.0034	0.36[0.173,0.732]*	0.0077	0.20[0.068,0.610]*	0.0071
PSMA5	0.91[0.433,1.913]	0.7964	0.83[0.414,1.648]	0.5668	0.84[0.239,2.959]	0.7694
DNMT1A	0.51[0.210,1.235]	0.1253	0.47[0.188,1.188]	0.1042	0.58[0.192,1.748]	0.3114
DNMT3A	0.69[0.355,1.357]	0.270	0.73[0.373,1.425]	0.3351	0.67[0.236,1.887]	0.4166
DNMT3B	0.23[0.092,0.568]*	0.0048	0.21[0.084,0.548]*	0.0040	0.21[0.075,0.591]*	0.0068
HDAC1	0.52[0.366,0.741]*	0.0009	0.60[0.425,0.851]*	0.0065	0.43[0.243,0.754]*	0.0058
HDAC2	0.32[0.143,0.714]*	0.0085	0.33[0.150,0.747]*	0.0109	0.24[0.069,0.838]*	0.0278
HDAC4	0.66[0.328,1.317]	0.2214	0.60[0.295,1.234]	0.1554	0.74[0.256,2.120]	0.5442

PBMC, peripheral blood mononuclear cells; CI, confidence interval; Relative expression ratio based on efficiency corrected delta delta Ct values presented as log values to the base 2, >1.5 indicates up regulation and ≤0.5 indicates down regulation in the experimental group compared to control group

* indicates statistically significant differential expression where P-value <0.05, P values calculated by paired t-tests

**Table 5:** mRNA expression in PBMC’s of melioidosis patients with diabetes (n = 20), melioidosis patients with regular alcohol consumption habits (n = 8) compared to healthy controls (n = 10) and sepsis controls (n = 10).

	Diabetic melioidosis vs Sepsis controls		Diabetic melioidosis vs healthy controls		Alcoholic melioidosis vs Sepsis controls	
Gene	Relative expression ratio [95% CI]	P value	Relative expression ratio [95% CI]	P value	Relative expression ratio [95% CI]	P value
HMGB1	0.30[0.150,0.601]*	0.0017	0.96[0.503,1.818]	0.8861	0.22[0.102,0.486] *	0.0009
IL6	0.97[0.341,2.760]	0.9525	1.70[0.627,4.624]	0.2828	0.44[0.123,1.585]	0.1869
IL8	0.42[0.164,1.088]	0.0718	1.63[0.703,3.778]	0.2414	0.27[0.078,0.936] *	0.0404
IL1β	0.67[0.223,2.024]	0.4528	1.16[0.477,2.803]	0.7345	0.60[0.155,2.313]	0.4320
IFNγ	0.97[0.544,1.743]	0.9245	1.48[0.905,2.431]	0.1111	0.66[0.361,1.211]	0.1657
TNFα	0.82[0.415,1.607]	0.5351	1.24[0.719,2.132]	0.4229	0.51[0.252,1.019]	0.0557
IL15	0.63[0.275,1.446]	0.2574	1.44[0.785,2.650]	0.2245	0.61[0.226,1.661]	0.3077
IL4	3.88[1.036,14.512]*	0.0471	1.69[0.069,41.297]	0.3921	2.86[0.833,9.802]	0.0893
TLR2	0.50[0.216,1.142]	0.0943	1.30[0.725,2.330]	0.3645	0.47[0.190,1.179]	0.1014
TLR4	0.42[0.158,1.129]	0.0818	1.11[0.582,2.101]	0.7512	0.37[0.130,1.042]	0.0586
MICB	0.37[0.201,0.688]*	0.0030	1.08[0.585,2.011]	0.7886	0.26[0.136,0.507] *	0.0006
HLADMB	0.80[0.462,1.379]	0.3995	0.97[0.586,1.599]	0.8932	0.76[0.396,1.470]	0.3939
PSMB2	0.29[0.141,0.603]*	0.0023	1.04[0.592,1.817]	0.8953	0.23[0.103,0.498] *	0.0011
PSME2	0.44[0.226,0.863]*	0.0197	1.45[0.876,2.394]	0.1418	0.35[0.164,0.738] *	0.0089
PSMB8	0.33[0.155,0.686]*	0.0053	1.00[0.535,1.854]	0.9885	0.29[0.129,0.631] *	0.0041
PSMA5	0.99[0.449,2.202]	0.9890	0.89[0.448,1.758]	0.7213	0.66[0.270,1.597]	0.3259
DNMT1A	0.53[0.213,1.324]	0.1622	0.70[0.368,1.319]	0.2549	0.40[0.151,1.047]	0.0606
DNMT3A	0.75[0.367,1.516]	0.4005	0.74[0.416,1.315]	0.2911	0.42[0.186,0.967] *	0.0425
DNMT3B	0.25[0.099,0.654]*	0.0084	1.26[0.679,2.321]	0.4451	0.17[0.067,0.446] *	0.0020
HDAC1	0.54[0.373,0.787]*	0.0025	0.84[0.588,1.200]	0.3220	0.44[0.302,0.636] *	0.0002
HDAC2	0.32[0.140,0.748]*	0.0112	0.77[0.416,1.429]	0.3939	0.23[0.095,0.553] *	0.0027
HDAC4	0.66[0.313,1.379]	0.2522	1.01[0.559,1.838]	0.9640	0.56[0.269,1.176]	0.1178

PBMC, peripheral blood mononuclear cells; CI, confidence interval; Relative expression ratio based on efficiency corrected delta delta Ct values presented as log values to the base 2, >1.5 indicates up regulation and ≤0.5 indicates down regulation in the experimental group compared to control group

* indicates statistically significant differential expression where P-value <0.05, P values calculated by paired t-tests

Septicaemic melioidosis patients showed a similar expression pattern as above, in addition to IL8 being down regulated compared to other sepsis cases (Table 3, Fig 1). IL8 down regulation was also seen in early acute melioidosis cases with less than 15 days of fever/clinical symptoms and antibiotic treatment. (Table 3, Fig 2). Melioidosis patients with regular alcohol consumption habits also expressed significant down regulation of IL8 compared to other sepsis cases (Table 5, Fig 3).

### Gene expression profile of Toll-like receptors (TLR’s)

TLR2 and TLR4 were significantly up regulated in sepsis caused by other pathogens compared to healthy controls. Melioidosis patients showed significantly down regulated expression of TLR4 while both TLR2 and TLR4 was down regulated in septicaemic melioidosis cases compared to sepsis caused by other pathogens (Table 2, Fig 1).

### Gene expression profile of genes associated with the antigen presentation pathway and cell-mediated immunity

MICB, PSMB2, PSMB8 and PSME2 were significantly up regulated in other sepsis cases compared to healthy controls while these target genes showed no significant differential expression in patients suffering from melioidosis compared to healthy controls. Therefore, the expression of these genes can be considered as down regulated in the melioidosis cohort including septicaemic melioidosis cases compared to other sepsis cases (Tables 2 and 3, Fig 1). This differential expression between melioidosis cases compared to sepsis controls was seen consistently, irrespective of other factors like duration of fever/clinical symptoms, antibiotic treatment and associated comorbidities (Tables 2–5, Figs 1–3).

### Gene expression profile of epigenetic modification factors

DNMT3B, HDAC1 and HDAC2 were significantly up regulated in other sepsis cases compared to healthy controls (Table 2, Fig 1). These epigenetic markers were significantly down regulated in melioidosis including septicaemic melioidosis cases compared to other sepsis cases (Tables 2 and 3, Fig 1). Our results also showed a consistent differential expression pattern of these epigenetic factors irrespective of other factors like duration of fever/clinical symptoms, antibiotic treatment and associated comorbidities (risk factors) (Tables 2–5, Figs 1–3). Melioidosis patients with a regular alcohol consumption habit also expressed significant down regulation of DNMT3A, apart from a similar differential expression of other markers compared to other sepsis cases (Table 5, Fig 3).

## Discussion

### Gene expression profile of cytokines

Our gene expression analysis of melioidosis patient samples did not reveal any statistically significant differential gene expression pattern in comparison with healthy controls (Table 2, Fig 1). The cytokine cascade events following *B*.*pseudomallei* infection has been studied in several animal models and show an up regulated mRNA expression of inflammatory cytokines such as IL6, IL12, IL10, IFNγ, TNFα and IL1β within 72 hours of infection[10, 11, 31, 32]. Studies have also shown an elevated level of expression of IL8, IL6, IL12, IL18, IL15, and IFNγ in plasma of melioidosis patients[12, 33]. Our findings are contrary to a published study showing an increased mRNA expression of inflammatory response genes such as IL6, IL15, IL10, IL4, IFNγ, TNFα and IL1β in melioidosis patients compared to healthy controls [9]. Majority of our samples in the melioidosis cohort, consisted of patients with greater than 10 days of fever/clinical symptoms and antibiotic treatment, in comparison to other studies which have sampled melioidosis patients in early acute phase, within 3 days of antibiotics treatment. This could have been the main reason for the contradicting result. We did not have adequate number of samples in early acute phase of melioidosis (n = 4) to see a statistically significant differential expression compared to healthy controls. Melioidosis patients display differential expression of several immune response genes compared to cases of sepsis resulting from other infections, indicating that the differential expression of these genes can be used as diagnostic marker for melioidosis.

IL4 was up regulated in the melioidosis patients, including the diabetic cohort, compared to other sepsis cases (Tables 2 and 5, Figs 1 and 3). IL4 plays a major role in B-cell activation and T-cell proliferation, thus acting as a key regulator of humoral and adaptive immunity. Its role as an anti-inflammatory cytokine participating in decreasing the production of Th1 cells and pro-inflammatory cytokines is suggestive of down regulation of inflammatory responses in melioidosis. Up regulated IL4 expression has been reported in melioidosis patients and acute melioidosis models [9, 13]. Thus, further investigations are currently being carried out on gene expression of IL4 and closely related anti-inflammatory cytokine IL13 in melioidosis patients.

IL8 was significantly down regulated in septicaemic melioidosis patients when compared to other sepsis cases, suggesting that it could be a marker of disease severity (Table 3, Fig 1). IL8 down regulation in early acute melioidosis cases (less than 15 days of fever/clinical symptoms and antibiotic treatment) was also seen compared to other sepsis cases (Table 3, Fig 2). A study using a human lung epithelial cell line showed that IL8 production upon *B*. *pseudomallei* infection was lower than cells infected with other gram negative bacteria which is in agreement with our findings [34]. Studies have also shown that *B*. *pseudomallei* can activate NF-κB and induce IL8 production without involving TLRs[35]. Increased level of plasma IL8, IL6 concentration being associated with mortality have also been reported[33]. Type 2 diabetes (T2D) has been reported to be a significant comorbidity associated to melioidosis, particularly septicaemic cases [36]. A study by Morris et al, showed significantly elevated levels of IL8 in plasma of diabetic cohorts compared to non-diabetics 3.5 hours after *B*. *pseudomallei* stimulation, suggesting a dysregulated immune response in T2D as underlying factor for susceptibility to melioidosis[37]. Thus IL8, a key mediator of innate immunity associated with inflammation, being down-regulated in early acute stages of melioidosis and in septicaemic cases needs to be investigated further with a larger sample pool, to get a better understanding of IL8’s role in disease severity.

HMGB1, classified as a late onset mediator of sepsis which may function as a pro-inflammatory and anti-bacterial factor [38, 39], was consistently down regulated in melioidosis patients irrespective of other confounding factors like comorbidities (risk factors) and duration of clinical symptoms and treatment, compared to other sepsis infection cases (Tables 2–5, Figs 1–3). Our findings also reveal a significantly up regulated expression of HMGB1 in other sepsis cases compared to healthy controls. HMGBI has been reported to show high levels of expression in plasma of melioidosis patients, particularly septicaemic melioidosis cases compared to other sepsis infections and has been associated with clinical severity and mortality[39]. Some studies have also shown that HMGB1, could induce Th2 type response, leading to increased production of anti-inflammatory cytokines like IL4, IL5 etc, while lowering the Th1 type response [40, 41]. HMGB1, plays a key role as an immune modulating factor and its potential role in cell mediated immune dysfunctions ought to be investigated further.

### Gene expression profile of Toll-like receptors

TLR2 and TLR4 did not exhibit a differential expression in melioidosis patients compared to healthy controls which is contrary to published studies showing up regulated expression[9, 16]. This contradiction, we believe is also due to variation in duration of clinical symptoms and antibiotic treatment in the studies. However, TLR4 is significantly up regulated in the sepsis cohort, compared to healthy controls (Table 2, Fig 1). As such bothTLR2 and TLR4 are down regulated in the septicaemic melioidosis cases compared to other sepsis infections (Table 2, Fig 1), suggesting poor pathogen recognition. Studies have indicated that LPS of *B*. *pseudomallei* signals *via* TLR2 as opposed to TLR4 being the main receptor for other gram-negative bacteria and TLR-mediated dysregulation of immune response plays a major role in disease pathogenesis [13, 16, 18]. HMGB1 shows a TLR4 dependent activity, with CD14 playing a key role in activation of TLR4 dependent signaling by HMGB1 has also been reported [42, 43].These correspond with our findings of a low level of expression of HMGB1, which plays a major role in activation of TLR4 mediated immune responses, hence down regulation of inflammatory and defense responses upon *B*. *pseudomallei* infection.

### Gene expression profile of genes associated with the antigen presentation pathway and cell mediated immunity

MICB, PSMB2, PSMB8 and PSME2 showed consistently low level of expression in melioidosis cohort irrespective of other factors like comorbidities (risk factors), duration of clinical symptoms and antibiotic treatment, compared to other sepsis controls (Tables 2–5, Figs 1–3). Low level of expression of these immune response genes which play a major role in antigen presentation and thereby cell mediated immunity, suggests altered defense responses during melioidosis or diminished proteasomal activity at the time of sample collection during later stages of infection. Our findings are contradicting with a similar study that showed differential gene expression pattern of proteasomes and other genes involved in MHC class I & II antigen presentation pathway, where genes PSMB2, PSME2, PSMB8, PSMA5 and HLADMB were over expressed in meliodosis patients compared to other sepsis cases [8].Variations in duration of clinical symptoms, administration of antibiotics at the time of sampling and associated comorbidities could have played a role in this contradicting result, while taking into consideration that the Pankla et al, 2009 study included samples mostly collected within 48 hours of hospitalization. It is also to be noted that the results of this 2009 study has not been independently verified.

### Gene expression profile of epigenetic modification factors

There have been extensive studies on the role of epigenetic factors and their association with several communicable and non-communicable diseases [19–21, 25]. DNA methylation, histone deacetylation and methylation are epigenetic modifications associated with a repressed chromatin state, which contributes to repression of gene transcription[44]. Studies have shown that DNMT3B, exhibits an inverse correlation between gene expression levels and DNA methylation levels[19]. A study by Bonsch et al, 2006 showed that genomic DNA hypermethylation was associated with lower mRNA levels of DNMT3B in patients with alcohol dependence [45]. Study by Zong et al 2015, showed that reduction of HDAC activity and expression, was associated with disease severity in smokers with chronic obstructive pulmonary disease (COPD)[20]. Epigenetic modifications are heavily influenced by practices such as smoking, alcohol dependence[19, 20], diseases such as diabetes, cardiovascular and kidney diseases [22, 23, 25, 46] which in turn could play a major role in disease pathogenesis and susceptibility.

A recent study on epigenetic changes in human host DNA following *B*. *pseudomallei* infection, revealed significant changes in DNA methylation in the vincity of genes involved in inflammatory responses, intracellular signaling, apoptosis and pathogen induced signaling, suggesting that DNA methylation changes could be altering gene transcription thus affecting key immune pathways [47]. High throughput gene expression analysis in melioidosis have not revealed strong association of epigenetic factors with *B*. *pseudomallei* infection [8]. However, extensive study in this area is required. While this study on whole-genome transcriptional profiles of septicaemic melioidosis and sepsis caused by other infections has revealed a transcriptional signature [8], those findings were never validated in an independent study. Therefore, we found it necessary to follow up on those studies, as well as investigate a unique set of target genes involved in epigenetic regulation to evaluate whether the expression of the epigenetics markers were deferentially regulated in melioidosis compared to sepsis caused by other infections. While those genes were not found to be deferentially expressed in whole-genome studies [8], given the relapse and reactivation of infection in susceptible groups, it was of interest to further investigate the transcription profiles of this set of genes involved in epigenetic modifications.

Our study investigated the differential expression of several epigenetic regulators in melioidosis. DNMT3B, responsible for DNA methylation, HDAC1 and HDAC2, responsible for histone deacetylation were significantly down regulated in melioidosis patients compared to other sepsis cases irrespective of confounding factors like duration of fever/clinical symptoms, antibiotic treatment and associated comorbidities (Tables 2–5, Figs 1–3). Thus, correlation between mRNA levels of DNMT’s and levels of methylation ought to be analyzed further. Our comparison of melioidosis patients with regular alcohol consumption habit (n = 8) and sepsis controls (n = 10) showed a similar expression pattern to the entire melioidosis cohort (n = 30), in addition to down regulated expression of DNMT3A and IL8 (Table 5, Fig 3). We could observe a significantly lower level of expression of the epigenetic factors in melioidosis cases compared to sepsis controls, while assessing alcohol consumption as a risk factor, indicating its confounding effect.

From our studies we find that epigenetic factors HDAC1, HDAC2 and DNMT3B show consistent differential expression compared to other sepsis cases, suggesting a role in disease susceptibility and pathogenesis. However, further studies such as DNA methylation arrays with a larger sample pool and further analysis of confounding comorbidities, is required to fully understand the role of epigenetic mechanisms in relation to pathogenesis of melioidosis.

### Limitation of study

The main limitation of our study was that the melioidosis patient samples were collected well after start of antibiotic treatment which may affect immunocompetant cells, which in turn affects the immune response genes investigated. Studies have shown that antibiotics like meropenem exert an immunomodulatory effect, affecting the production of some cytokines in PBMC’s [48]. This may have been the main reason, as to why we could not see any significant differential expression of some key inflammatory response cytokines such as IFNγ, especially between the melioidosis and healthy cohorts, while similar studies have sampled within 3 days of antibiotic treatment. Duration of clinical symptoms ranged from >10 days to >90 days and duration of antibiotics treatment ranged from 1 day to >30 days at the time of blood collection for all the melioidosis samples. Since our sample collection was nationwide, duration between patient identification/ disease confirmation and sampling was substantial due to logistical issues. Thus, due to wide range of duration of infection and limited number of samples with ≤15 days of fever (n = 4) we could not evaluate statistically significant differential expression of inflammatory genes during the early stages of infection. Melioidosis is severely under-reported and under-diagnosed in Sri Lanka and most patients who get hospitalized and diagnosed of the disease, are already in a later stage of infection. This makes it very difficult to carryout investigations with patient samples within an early stage of infection and or anti-microbial therapy. While its important to gather information on expression levels of host factors during early acute phase of infection, it is also imperative to have some understanding on expression levels of important host genes during later stages of infection like in our study, which may be useful to monitor antibiotic treatment regimes. As we see diabetes as a major comorbidity in our experimental cohort, we analyzed our data to see if there was any significant differential expression between diabetic melioidosis cases and non-diabetic melioidosis cases. The gene expression between these two groups were comparable and we could not find any statistically significant differential expression due to diabetes (S3 Table). Therefore, our results show a consistent differential expression of HMGB1, MICB, PSMB8, PSMB2, PSME2, HDAC1, HDAC2 and DNMT3B when compared to other sepsis cases, irrespective of comorbidities (risk factors), duration of fever/clinical symptoms and antibiotic treatment, primarily due to melioidosis infection. We also note that gene expression analyses of blood leukocytes only provide insight in immune pathways regulated at mRNA level in circulating cells, hence we look to expand our future study on a proteomic level as well. We also take note of the limitation of IHA test used in this study for diagnosis of melioidosis. Though the sensitivity and specificity of IHA test is limited, it has been used worldwide as a laboratory method for melioidosis diagnosis. The bacterial loads of patient samples are not always high for culture positive results, with general antibiotic treatment playing an intervening role. Hence IHA test results were included, as they serve as a useful reference for melioidosis diagnosis.

### Conclusion

Our findings did not show significant differential expression among the immune response genes and the epigenetic modifiers in melioidosis cases and the healthy controls. However, our study indicates differential expression in inflammatory responses, defense responses and epigenetic factors during melioidosis compared to other cases of sepsis, thus differential gene expression among the genes under investigation that can distinguish melioidosis cases from other sepsis infections. These findings suggest that the differentially expressed genes during melioidosis should be validated during different stages of infection for their potential as markers of disease diagnosis and for therapeutic intervention. Thus, our future studies shall be aimed at studying gene expression profiles in early and late acute phases of melioidosis and also looking into susceptible groups for further study on disease severity. Studies would also be broadened into the four areas showing differential gene expression pattern- key cytokines, antigen presentation pathways, toll-like receptor signaling pathways and epigenetic chromatin modifying enzymes. Further investigations to confirm these findings in a larger cohort is needed, which may validate the potential of these differentially expressed genes to serve as disease biomarkers that could pave the way for novel diagnostic and therapeutic approaches for melioidosis intervention.

## Supporting information

S1 Data Clinical data supplementary file. (XLSX)

S1 Table List of gene targets investigated, their full name and biological role. (DOCX)

S2 Table Primer details for Gene expression analysis. (DOCX)

S3 Table mRNA expression in PBMC’s of diabetic melioidosis cases (n = 20) compared to non-diabetic melioidosis cases (n = 10). (DOCX)
