# Supplementary material for: Host gene expression analysis in Sri Lankan melioidosis patients
Source: PLoS Negl Trop Dis. 2017 Jun 19;11(6):e0005643. doi: 10.1371/journal.pntd.0005643 (PMC5498071; doi:10.1371/journal.pntd.0005643)
Supplement: S1 Table — (DOCX) [file pntd.0005643.s002.docx]

**S1: List of gene targets investigated**

| **Abbreviated name** | **Full Name** | **Biological Role** |
| --- | --- | --- |
| GAPDH | Glyceraldehyde 3-phosphate dehydrogenase | Involved in several metabolic processes such as glycolysis |
| 18SrRNA | 18S ribosomal RNA | Structural RNA and basic component of eukaryotic cells |
| PLCE1 | 1-Phosphatidylinositol-4,5-bisphosphate phosphodiesterase epsilon-1 | Involved in intracellular responses involving cell growth and differentiation |
| IL1β | Interleukin 1 beta | Pro-inflammatory cytokine, important mediator of the inflammatory response, involved in a variety of cellular activities, including cell proliferation, differentiation and apoptosis. |
| IL4 | Interleukin 4 | Anti-inflammatory cytokine that induces differentiation of naive helper T cells (Th0 cells) to Th2 cells, decreasing production of Th1 cells. It is a key regulator in humoral and adaptive immunity playing a major role in stimulation of activated B-cell and T-cell proliferation. |
| IL6 | Interleukin 6 | Pro-inflammatory cytokine and an anti-inflammatory myokine. It is secreted by T cells and macrophages to stimulate immune response |
| IL8 | Interleukin 8 | Chemokine associated with inflammation, induces chemotaxis in target cells, mainly involved in neutrophil recruitment and degranulation. |
| IL10 | Interleukin 10 | Anti-inflammatory cytokine involved in immune regulation and inflammation. It down regulates the expression of Th1 cytokines, MHC class II antigens, and co-stimulatory molecules on macrophages. It also enhances B cell survival, proliferation, and antibody production. IL-10 can block NF-κB activity, and is involved in the regulation of the JAK-STAT signaling pathway. |
| IL12 | Interleukin 12 | Pro-inflammatory cytokine involved in the differentiation of naive T cells into Th1 cells. It is involved in stimulation and growth T cells and in production of interferon-gamma (IFN-γ) and tumor necrosis factor-alpha (TNF-α) from T cells and natural killer (NK) cells. It reduces IL-4 mediated suppression of IFN-γ. |
| IL15 | Interleukin 15 | Pro-inflammatory cytokine which regulates T and natural killer (NK) cell activation and proliferation. |
| IL18 | Interleukin 18 | Pro-inflammatory cytokine involved in inflammation and cell-mediated immunity along with IL12 |
| CCL5 | Chemokine (C-C motif) ligand 5. Also known as RANTES (regulated on activation, normal T cell expressed and secreted). | Chemokine, which is chemotactic for T cells, eosnophils and basophils, plays an active role in recruiting leukocytes into inflammatory sites |
| IFNγ | Interferon gamma | Pro-inflammatory cytokine that is critical for innate and adaptive immunity against viral, some bacterial and protozoal infections. It is an important activator of macrophages and inducer of Class II major histocompatibility complex (MHC) molecule expression. |
| TNFα | Tumor necrosis factor alpha | Pro-inflammatory cytokine involved in systemic inflammation and immune regulation |
| HMGB1 | High mobility group box 1 protein, also known as high-mobility group protein 1 (HMG-1) | Cytokine mediator of inflammation secreted by activated macrophages and monocytes |
| TLR2 | Toll-like receptor 2 | Plays a fundamental role in pathogen recognition and activation of innate immunity. This gene is expressed abundantly in peripheral blood leukocytes and mediates host response to gram-positive bacteria. |
| TLR4 | Toll-like receptor 4 | Plays a fundamental role in pathogen recognition and activating the innate immune system. It is well-known for recognizing lipopolysaccharide (LPS), a component present in many gram-negative bacteria, thus mediates its host responses |
| MICB | MHC class I polypeptide-related sequence B | Heavily glycosylated protein which is a ligand for the NKG2D type II receptor. Binding of the ligand activates the cytolytic response of natural killer (NK) cells, CD8 alpha beta T cells, and gamma delta T cells which express the receptor. |
| PSMB8 | Proteasome subunit beta type-8 also known as 20S proteasome subunit beta-5i | Forms a pivotal component for the Ubiquitin-Proteasome System (UPS) involved in protein ubiquitination and subsequent proteolysis and degradation which are important mechanisms in the regulation of the cell cycle, cell growth and differentiation, gene transcription, signal transduction and apoptosis. During the antigen processing for the major histocompatibility complex (MHC) class-I, the proteasome is the major degradation machinery that degrades the antigen and present the resulting peptides to cytotoxic Tcells. |
| PSMB2 | Proteasome subunit beta type-2 also known as 20S proteasome subunit beta-4 | Forms a pivotal component for the UPS involved in protein ubiquitination and subsequent proteolysis and degradation which are important mechanisms in the regulation of the cell cycle, cell growth and differentiation, gene transcription, signal transduction and apoptosis. It is also involved in processing of class I MHC peptides |
| PSME2 | Proteasome activator complex subunit 2 | Process class I MHC peptides |
| PSMA5 | Proteasome subunit alpha type-5 also known as 20S proteasome subunit alpha-5 | Process class I MHC peptides |
| HLADMB | HLA class II histocompatibility antigen, DM beta chain | Plays a central role in the peptide loading of MHC class II molecules by helping to release the CLIP (class II-associated invariant chain peptide) molecule from the [peptide](https://en.wikipedia.org/wiki/Peptide) [binding site](https://en.wikipedia.org/wiki/Binding_site), thus playing a major role in MHC class II antigen presentation pathway |
| DNMT1A | DNA methyltransferase 1A | Enzyme catalyzes the transfer of methyl groups to specific CpG structures in DNA (DNA methylation). Considered to be the key maintenance methyl transferase in mammals. predominantly methylates hemi methylated CpG di-nucleotides in the mammalian genome. |
| DNMT3A | DNA (cytosine-5)-methyl transferase 3A | DNA methyl transferase responsible for de novo DNA methylation. |
| DNMT3B | DNA (cytosine-5-)-methyl transferase 3 beta | DNA methyl transferase responsible for de novo DNA methylation. |
| HDAC1 | Histone deacetylase 1 | Class I histone deacetylase, playing a key role in the regulation of eukaryotic gene expression |
| HDAC2 | Histone deacetylase 2 | Class I histone deacetylase, playing a key role in transcriptional regulation and regulation of eukaryotic gene expression |
| HDAC4 | Histone deacetylase 4 | Class II histone deacetylase, playing a key role in transcriptional regulation and regulation of eukaryotic gene expression |
