# Supplementary material for: Host gene expression analysis in Sri Lankan melioidosis patients
Source: PLoS Negl Trop Dis. 2017 Jun 19;11(6):e0005643. doi: 10.1371/journal.pntd.0005643 (PMC5498071; doi:10.1371/journal.pntd.0005643)
Supplement: S2 Table — (DOCX) [file pntd.0005643.s003.docx]

**S2: Primer details for Gene expression analysis**

| Primer Name | Forward and Reverse Sequence | Accession number | Product  Size |
| --- | --- | --- | --- |
| GAPDH^a^ | TGACAACTTTGGTATYCGTGGAAGG  AGGCAGGGATGATGTTCTGGAGAG | NM_001289746.1 | 134 bp |
| 18SrRNA^a^ | GCTTAATTTGACTCAACACGGGA  AGCTATCAATCTGTCAATCCTGTC | NR_003286.2 | 69 bp |
| PLCE1^b^ | GCCCAAAGCAAGTGGAAAGG  TCTTCACCTGGGTTAAACATGC | XM_011539850.2 | 700 bp |
| IL8 | CAGAGACAGCAGAGCACACA  GGCAAAACTGCACCTTCACA | NM_000584.3 | 158 bp |
| MICB | CACCCAGGCTGCAGTTCACT  CGGGAGTCTGAGGTACGAGAA | NM_001289160.1 | 88 bp |
| PSMB8 | GATCTCCAGAGCTCGCTTTA  GTTCACCCGTAAGGCACTAA | NM_148919.3 | 200 bp |
| CCL5 | CCATGAAGGTCTCCGCGGCAC  CCTAGCTCATCTCCAAAGAG | NM_001278736.1 | 361 bp |
| IL6 | GGTACATCCTCGACGGCATCT  GTGCCTCTTTGCTGCTTTCAC | XM_011515390.2 | 88 bp |
| IL18 | GCTTGAATCTAAATTATCAGTC  CAAATTGCATCTTATTATCATG | XM_011542806.2 | 335 bp |
| DNMT1A | ACCGCTTCTACTTCCTCGAGGCCTA  GTTGCAGTCCTCTGTGAACACTGTGG | NM_001318731.1 | 335 bp |
| DNMT3B | AATGTGAATCCAGCCAGGAAAGGC  ACTGGATTACACTCCAGGAACCGT | XR_936512.2 | 191 bp |
| HDAC1 | CCAAGTACCACAGCGATGAC  TGGACAGTCCTCACCAACG | XM_011541309.2 | 110 bp |
| HDAC2 | TGAAGGAGAAGGAGGTCGAA  GGATTTATCTTCTTCCTTAACGTCTG | XM_017010799.1 | 124 bp |
| DNMT3A | CGTTGGCATCCACTGTGAATGA  TTACACACACGCAAAATACTCCTT | XM_017003527.1 | 329 bp |
| IFNγ | CCAACGCAAAGCAATACATGA  CCTTTTTCGCTTCCCTGTTTTA | NM_000619.2 | 79 bp |
| TNFα | GGA GAA GGG TGA CCG ACT CA  CTG CCC AGA CTC GGC AA | NM_000594.3 | 70 bp |
| IL1β | GCAAGGGCTTCAGGCAGGCCGCG  GGTCATTCTCCTGGAAGGTCTGTGGGC | XM_017003988.1 | 96 bp |
| IL4 | ACTTTGAACAGCCTCACAGAG  TTGGAGGCAGCAAAGATGTC | NM_000589.3 | 74 bp |
| IL15 | GTCTTCATTTTGGGCTGTTTCAGT  CCTCACATTCTTTGCATCCAGATTCT | NR_037840.2 | 316 bp |
| TLR2 | GGGTCATCATCAGCCTCTCC  AGGTCACTGTTGCTAATGTAGGTG | XM_011532216.2 | 181 bp |
| TLR4 | CAGAGTTGCTTTCAATGGCATC  AGACTGTAATCAAGAACCTGGAGG | NM_138557.2 | 282 bp |
| PSME2 | GGGAATGAGAAAGTCCTGTCC  TCAATCTTGGGGATCAGGTG | XM_006720213.1 | 113 bp |
| IL12 | CCAAGAACTTGCAGCTGAAG  TGGGTCTATTCCGTTGTGTC | NM_002187.2 | 355 bp |
| HDAC4 | GAGAGACTCACCCTTCCCG  CCGGTCTGCACCAACCAAG | XM_017005395.1 | 240 bp |
| PSMA5 | AAGCCCATGTTGCTTTTGGG  GGCGAACGGACATAGGCTAA | [NM_001199774.1](https://www.ncbi.nlm.nih.gov/nucleotide/315138984?report=genbank&log$=nucltop&blast_rank=1&RID=E113VTMK013) | 112 bp |
| PSMB2 | AGAGGGCAGTGGAACTCCTT  AGGTTGGCAGATTCAGGATG | NM_001199779.1 | 72 bp |
| HMGB1 | ACATCCAAAATCTTGATCAGTTA  AGGACAGACTTTCAAAATGTTT | NM_001313893.1 | 122 bp |
| HLADMB | ATGTGAAATCCTTTGGAGTCCCA  GGCATCTTTACAGAGCAGAGCAT | NM_002118.4 | 145 bp |

^a^GAPDH and 18srRNA primers are house keeping genes. ^b^PLCE1 primer pair tested to amplify 700bp genomic region of PLCEI is used as genomic DNA control.
