# Supplementary material for: Host gene expression analysis in Sri Lankan melioidosis patients
Source: PLoS Negl Trop Dis. 2017 Jun 19;11(6):e0005643. doi: 10.1371/journal.pntd.0005643 (PMC5498071; doi:10.1371/journal.pntd.0005643)
Supplement: S3 Table — (DOCX) [file pntd.0005643.s004.docx]

**S3: mRNA expression in PBMC’s of diabetic melioidosis cases (n=20) compared to non-diabetic melioidosis cases (n=10)**

|  | **Experimental: Diabetic Meliodosis ( N = 20 )** | | |
| --- | --- | --- | --- |
|  | **Control: Non/Diabetic Meliodosis ( N = 10 )** | | |
| **Gene Target** | **Relative Expression Ratio** | **95% Confidence Limit** | **P-Value** |
| DNMT1A | 1.238 | 0.468 , 3.275 | 0.642 |
| DNMT3A | 1.44 | 0.597 , 3.475 | 0.3917 |
| DNMT3B | 1.568 | 0.824 , 2.983 | 0.1559 |
| HDAC1 | 1.351 | 0.669 , 2.727 | 0.3749 |
| HDAC2 | 1.446 | 0.534 , 3.914 | 0.4384 |
| HDAC4 | 1.136 | 0.429 , 3.009 | 0.7828 |
| HLADMB | 1.425 | 0.569 , 3.574 | 0.4192 |
| HMGB1 | 1.498 | 0.556 , 4.037 | 0.4008 |
| IFNγ | 1.549 | 0.641 , 3.745 | 0.3013 |
| IL15 | 1.378 | 0.435 , 4.367 | 0.55 |
| IL1B | 0.615 | 0.152 , 2.486 | 0.4655 |
| IL4 | 0.914 | 0.016 , 51.946 | 0.8896 |
| IL6 | 1.078 | 0.274 , 4.246 | 0.9098 |
| IL8 | 0.944 | 0.203 , 4.378 | 0.9365 |
| MICB | 1.404 | 0.585 , 3.371 | 0.4248 |
| PSMA5 | 1.61 | 0.674 , 3.846 | 0.2667 |
| PSMB2 | 1.443 | 0.673 , 3.094 | 0.3253 |
| PSMB8 | 1.548 | 0.595 , 4.023 | 0.3448 |
| PSME2 | 1.617 | 0.688 , 3.803 | 0.251 |
| TLR2 | 1.418 | 0.415 , 4.852 | 0.5469 |
| TLR4 | 1.507 | 0.412 , 5.512 | 0.5046 |
| TNFα | 0.949 | 0.337 , 2.672 | 0.9139 |
